# Spectrum of human *Pasteurella* species infections in tropical Australia

**DOI:** 10.1371/journal.pone.0281164

**Published:** 2023-01-31

**Authors:** Michelle Mahony, Dimitrios Menouhos, Jann Hennessy, Robert W. Baird

**Affiliations:** Northern Territory Department of Health, Territory Pathology, Darwin, Northern Territory, Australia; University of Illinois Urbana-Champaign, UNITED STATES

## Abstract

**Background:**

Acquired zoonotic infections with *Pasteurella* bacterial species have a wide clinical spectrum of disease from invasive infections to localised bite-wound infections.

**Methods:**

This study reviewed the spectrum of the demographic, clinical, temporal, and microbiological trends of laboratory confirmed *Pasteurella* species infections presenting to a single-centre tropical tertiary hospital over a twenty-year period.

**Results:**

195 episodes from 190 patients were included. 51.3% patients were female, and 20.5% Aboriginal or Torres Strait Islander peoples. Crude incidence of *Pasteurella* spp. infections increased from 1.5 per 100,000 population in 2000, to 11.4 per 100,000 population in 2021. There were 22 (11.3%) bloodstream infections, 22 (11.3%) invasive, 34 (17.4%) deep local, 98 (50.2%) superficial infections, and 19 (9.7%) other or unknown. Adults over 65 years of age accounted for the majority of bacteraemias (63.7%). More severe infections, including bacteraemia, invasive and deep local infections, were more common in lower limb infections and in those with underlying comorbidities. Animal contact with cats was more common in bloodstream infections (36.4%), but dog bites more common in invasive, deep local and superficial infections. 30-day all-cause mortality was low at 1.0%. *Pasteurella multocida* was most commonly identified (61.1%), but *P*. *canis*, *P*. *dagmatis*, and other *Pasteurella* infections were also noted. 67.7% of specimens were polymicrobial, with other significant organisms being *Staphylococcus aureus*, *Streptococcus pyogenes*, Group G Streptococcus and *Pseudomonas aeruginosa*.

**Conclusion:**

*Pasteurella* species remain clinically important pathogens, with the ability to cause severe and invasive infections with associated morbidity. Presentations to hospital are becoming more common, and the polymicrobial nature of bites wounds has implications for empiric antibiotic guidelines.

## Introduction

*Pasteurella* species are oxidase and catalase positive Gram-negative coccobacilli, found as part of the normal flora of the oral and gastrointestinal tracts of domestic and wild animals. They are associated with veterinary infections and acquired zoonotic infections in humans. The human disease spectrum ranges from the more common bite wound cellulitis [1], invasive infections such as bacteraemia [2], respiratory disease (particularly in patients with underlying pulmonary disease), osteoarticular infections and genitourinary infections [3].

Animal bites, from dogs and cats in particular, are the classic human *Pasteurella* spp. associated infection [1,4]. Almost two-thirds of Australian households have a pet, including an estimated 5.1 million dogs and 3.8 million cats nationally in 2019 [5]. It is estimated there are over 100,000 dog bites per year in Australia [4]. However, laboratory confirmed *Pasteurella* spp. infections are relatively uncommon, and are not generally notifiable in Australia. Published studies have focused on the more serious invasive infections [2,6] with appreciable mortality, or on dog bite presentations to the emergency department [4,7–9].

Internationally, *Pasteurella multocida subsp multocida* is the most commonly isolated of the *Pasteurella* spp. associated with human infection (2). Published epidemiological findings are relatively infrequent, though in England and Wales there are around 600 laboratory confirmed cases reported in humans each year, of which about 70% are due to *P*. *multocida*. Most of these cases occur in people over 50 years of age. In 2013, there were 714 laboratory confirmed cases reported in the United Kingdom (6). Other studies have focused on invasive disease rather than the entire spectrum of disease [10–12].

Understanding the entire spectrum of disease allows for a balanced perspective on the severity of *Pasteurella* spp. infections, risk of complications and appropriate management strategy. In this present study we reviewed the demographic, clinical, temporal, and microbiological trends of laboratory confirmed *Pasteurella* spp. infections presenting to a single-centre tropical tertiary hospital over a twenty-year period.

## Materials and methods

The human research ethics committee at Menzies School of Health Research approved this study and granted a waiver of individual consent (HREC-2022-4234). We conducted a single-centre retrospective study of patients with laboratory-confirmed *Pasteurella* spp. infections between 2000 and 2021, presenting to the only tertiary referral hospital in the Top End. The Top End of the Northern Territory in Australia encompasses over 245 000 km^2^, with an estimated population of 200 610 in 2020 [13], 26.5% of which identify as Aboriginal or Torres Strait Islander [14]. There is a tropical climate with three distinct seasons: monsoon (January – April), dry (May – August) and “build up” to the wet season (September – December).

A single laboratory service provides microbiology services to the public hospitals in the Top End. The laboratory uses CLSI methodology for identification, susceptibility testing and reporting of microbiological results, with the Biomerieux VITEK MS and VITEK 2 used for identification to the species level. No additional typing of *Pasteurella* spp. isolates were performed, in keeping with routine laboratory practice for clinical specimens. All hospital encounters were included (inpatient, outpatient and emergency department presentations). Positive cultures were identified through the laboratory information system, Intersystems TrakCare-Lab 2016 [15].

Repeat isolates were classified in the same clinical episode if collected within seven days. Polymicrobial infections were defined as specimens with bacteria other than *Pasteurella* spp. noted on Gram stain, or isolated on culture.

All patients with a positive blood culture were included in the bloodstream infection group, even if they had evidence of distant foci of infection. Invasive infections included isolation from an otherwise sterile site or distant infection, including osteoarticular specimens, pulmonary isolates and peritoneal fluid. Deep local infections were defined as localised wound infection with secondary abscess formation, underlying tendon involvement, or evidence of necrotising skin infection. Superficial infections included localised bite wound infections and cellulitis.

Electronic medical records, where available, were retrospectively reviewed by a single author for demographic, clinical, surgical and microbiological data.

Postcodes were classified as outer regional, remote and very remote, as per the Australian Statistical Geography Standards (ASGS) [16], and incidence calculated using the Australian Bureau of Statistics Estimated Resident Population, 2021 [17].

Statistical analysis was conducted using Stata/SE 17.0. In the descriptive analysis, categorical variables were expressed as numbers (percentage), and continuous variables as median (and interquartile range) depending on the data distribution. Continuous variables were compared using Kruskal-Wallis rank test. Categorical variables were compared using Pearson’s chi-square test or Fischer’s exact test when required. Mapping was performed using QGIS-LTR 3.22 and Open Street View.

## Results

There were 195 clinical episodes identified from 190 patients. 44 infections (23%) were classified as bacteraemia or invasive at presentation. Three patients had two separate clinical episodes. One patient had three presentations at six-month intervals with *Pasteurella* spp. bacteraemia secondary to scratches from his cat.

**Fig 1:**
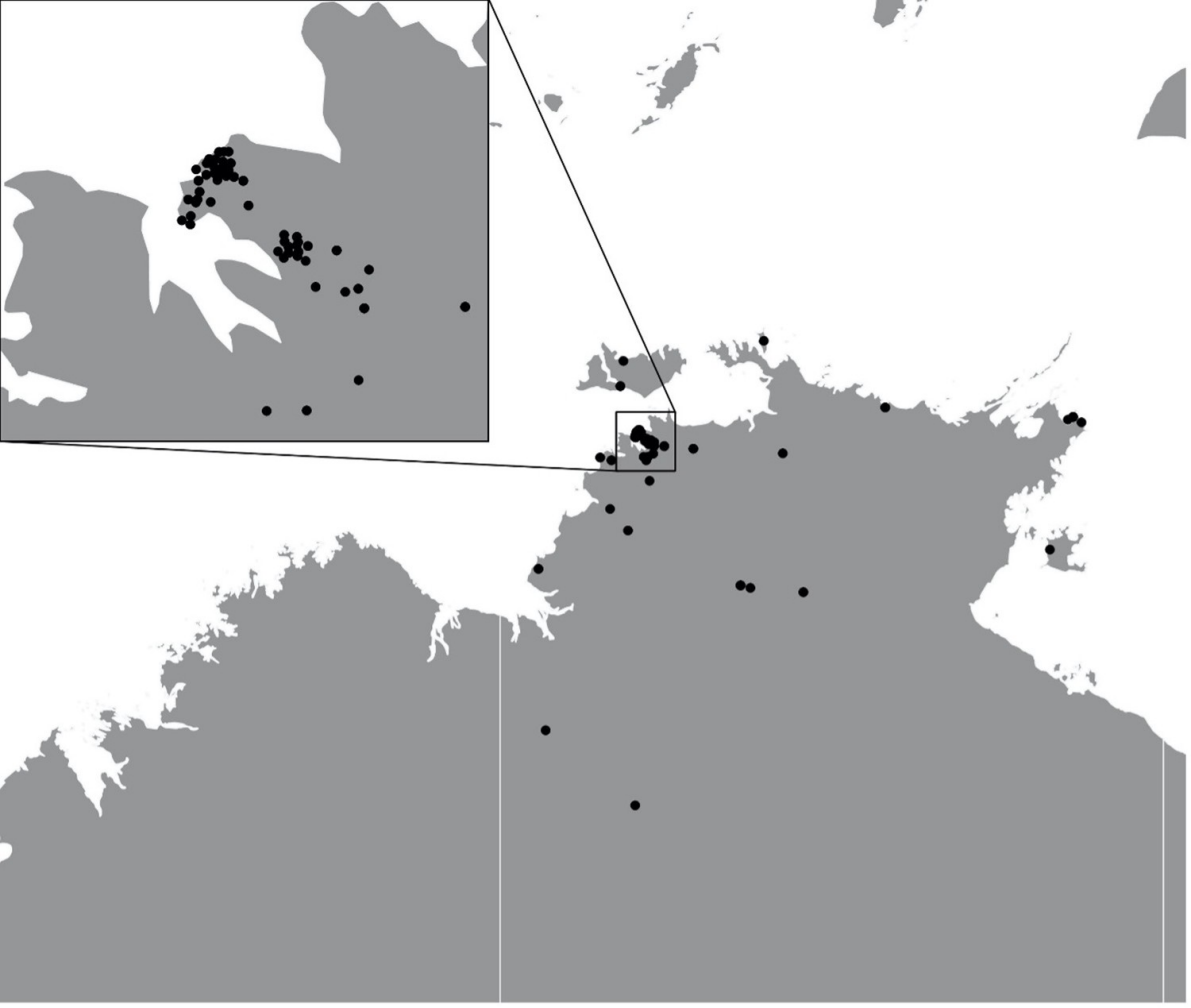
Geographic distribution of residential addresses of patients presenting with *Pasteurella* spp. infection.

The demographics of the patients are outlined in Table 1. 100 (51.3%) of the study population were female. Twenty-one (10.8%) were children under 16 years of age, and 43 (22.1%) aged over 65 years. Forty (20.5%) identified as Aboriginal or Torres Strait Islander; they had a younger median age than non-Aboriginal or Torres Strait Islander patients (median age 41.5 years vs 49.7 years, *p = 0*.*010*) and were more likely to live in remote or very remote settings (50% vs 15.5%, *p < 0*.*001*). Cases were centred geographically around urban areas and regional centres (Fig 1), in line with overall population density [13].

**Table 1:** Demographics of patients with *Pasteurella* spp. infections in the Top End from 2000-2021.

		Aboriginal or Torres Strait Islander, n (%)	Not Aboriginal or Torres Strait lslander, n (%)	Total, n (%)	P value
**Number**		40 (20.5)	155 (79.5)	195 (100)	
**Age (years), median (IQR)**		41.5 (28.9–50.0)	51.5 (30.4–67.3)	49.7 (30.0–62.6)	*P = 0*.010 *
**Age**	**< 16 years**	5 (12.5)	16 (10.3)	21 (10.8)	*P = 0*.045 *
	**16-65 years**	32 (80.0)	99 (63.9)	131 (67.2)	
	**> 65 years**	3 (7.5)	40 (25.8)	43 (22.0)	
**Sex**	**Male**	22 (55.0)	73 (47.1)	95 (48.7)	*P = 0*.*373*
	**Female**	18 (45.0)	82 (52.9)	100 (51.3)	
**Residence**	**ASGS 3 (Outer Regional)**	20 (50.0)	120 (77.4)	140 (71.8)	*P < 0*.001 *
	**ASGS 4 (Remote)**	7 (17.5)	19 (12.3)	26 (13.3)	
	**ASGS 5 (Very Remote)**	13 (32.5)	5 (3.2)	18 (9.2)	
	**Interstate**	0	11 (7.1)	11 (5.6)	
**Clinical category**	**Bloodstream**	3 (7.5)	19 (12.3)	22 (11.3)	*P = 0*.001 *
	**Invasive**	11 (27.5)	11 (7.1)	22 (11.3)	
	**Deep local**	7 (17.5)	27 (17.4)	34 (17.4)	
	**Superficial local**	15 (37.5)	83 (53.5)	98 (50.2)	
	**Other**	3 (7.5)	2 (1.3)	5 (2.6)	
	**Unknown**	1 (2.5)	13 (8.4)	14 (7.2)	

**Fig 2:**
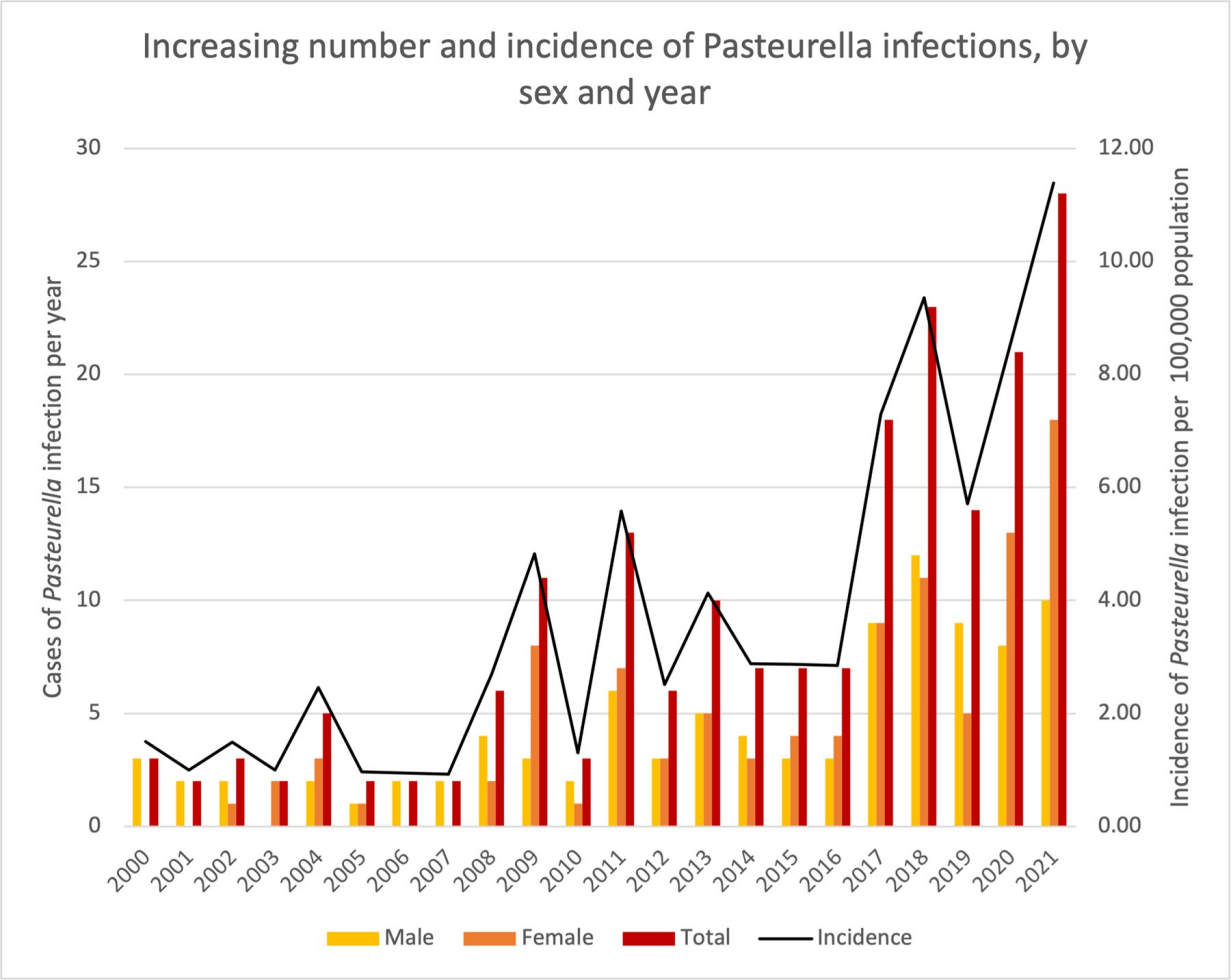
Increasing clinical episodes and incidence of *Pasteurella* spp. infections, 2000-2021.

The number of clinical episodes each year increased over the study period (Fig 2), with a median of three cases per year in 2000-2010, and 13 in 2011-2021 (*p < 0*.*001*). The greatest number was 28 cases in 2021. Crude incidence of *Pasteurella* spp. infections increased from 1.5 per 100,000 population in 2000, to 11.4 per 100,000 population in 2021. Crude incidence of bacteraemia increased from 0.5 per 100,000 population in 2000, to 1.2 per 100,000 population in 2021. There were no differences in seasonality.

### Clinical characteristics and outcomes

Complete clinical information was available for 137 of 195 (70.3%) episodes. Reasons for incomplete clinical information included incomplete electronic documentation, or subsequent management in a private hospital. Complete documentation was more common in the later years of the study period, as electronic prescribing and discharge summaries became more widely adopted, but documentation did not differ based on demographics or type of presentation. Admission or attendance information was available for all cases managed in the public health system alone.

Clinical characteristics are outlined in Table 2. There were 22 clinical episodes of bacteraemia, including patients with cellulitis (9; 40.9%), septic arthritis (2; 9.1%), infective endocarditis (1; 4.5%), pneumonia (1; 4,5%), and odontogenic infection (1; 4,5%). Associated lower limb infections (14, 63.7%) were the most common. Six patients with bacteraemia had no distant foci of infection found; clinical information was incomplete in two. Patients with bacteraemia were most commonly aged over 65 years (14; 63.7%) with a substantial proportion (5; 22.7%) over 85 years.

**Table 2:** Clinical characteristics of patients with *Pasteurella* spp. infections in the Top End from 2000-2021.

		Bloodstream (n = 22)	Invasive (n = 22)	Deep local (n = 34)	Superficial local (n = 98)	Other ^a^ (n = 5)	Unknown (n = 14)	Total (n = 195)	P value
**Age, median (IQR)**		71.7 (58.4 – 82.9)	54.5 (42.8 – 62.7)	46.7 (22.6 – 62.6)	45.8 (28.6 – 56.0)	49.9 (28.0 – 55.6)	32.5 (25.9 – 60.0)	49.7 (30.0 – 62.6)	*P < 0*.001 *
**Age group**	**< 16 years**	1 (4.5)	1 (4.5)	4 (11.8)	12 (12.2)	1 (20.0)	2 (14.3)	21 (10.8)	*P = 0*.002 *
	**16 – 65 years**	7 (31.8)	16 (72.8)	23 (67.6)	71 (72.5)	3 (60.0)	11 (78.6)	131 (67.2)	
	**> 65 years**	14 (63.7)	5 (22.7)	7 (20.6)	15 (15.3)	1 (20.0)	1 (7.1)	43 (22.0)	
**Sex**	**Male**	17 (77.3)	12 (54.5)	14 (41.2)	42 (42.9)	2 (40.0)	8 (57.1)	95 (48.7)	*P = 0*.*071*
	**Female**	5 (22.7)	10 (45.5)	20 (58.8)	56 (57.1)	3 (60.0)	6 (42.9)	100 (51.3)	
**Animal contact**	**Dog**	4 (18.2)	4 (18.2)	15 (44.1)	53 (54.1)	0	0	82 (42.1)	*P < 0*.001 *
	**Cat**	8 (36.4)	0	12 (35.3)	39 (39.8)	0	3	62 (31.8)	
	**Other**	0	0	1 (2.9)	0	0	0	1 (0.5)	
	**Unknown**	10 (45.4)	18 (81.8)	6 (17.7)	6 (6.1)	5 (100.0)	5 (100.0)	50 (25.6)	
**Location of infection**	**Upper limb**	0	2 (9.1)	9 (26.5)	48 (49.0)	0	2 (14.3)	61 (31.2)	*P < 0*.001 *
	**Lower limb**	14 ^b^ (63.6)	6 (27.3)	22 (64.7)	40 (40.8)	0	8 (57.1)	90 (46.1)	
	**Head and neck**	1 (4.5)	1 (4.5)	1 (2.9)	5 (5.1)	0	0	8 (4.1)	
	**Pneumonia**	1 (4.5)	11 (50.0)	0	0	0	0	12 (6.2)	
	**Other**	1 (4.5)	2 (9.1)	2 (5.9)	2 (2.0)	5 (100.0)	0	12 (6.2) ^c^	
	**Not stated**	5 (22.7)	0	0	3 (3.1)	0	4 (28.6)	12 (6.2)	
**Number of specimens with *Pasteurella* spp. isolated**	**1**	13 (59.1)	12 (54.5)	14 (41.2)	82 (83.7)	3 (60.0)	13 (92.9)	137 (70.3)	*P < 0*.001 *
	**2**	7 (31.8)	6 (27.3)	17 (50.0)	14 (14.3)	2 (40.0)	1 (7.1)	47 (24.1)	
	**3 or more**	2 (9.1)	4 (18.2)	3 (8.8)	2 (2.0)	0	0	11 (5.6)	
**Comorbidities**		20/21 (95.2) ^d^	14/19 (73.7) (95.2) ^d^	21/33 (63.6) (95.2) ^d^	56/96 (58.3) (95.2) ^d^	3/3 (100.0) (95.2) ^d^	1/1 (95.2) ^d^	99/173 (57.2) (95.2) ^d^	*P < 0*.001 *

^a^ includes three urine cultures, one ear swab and one eye swab.

^b^ includes bacteraemia and cellulitis or septic arthritis of lower limb, even if *Pasteurella* spp. was only isolated from blood.

^c^ one infective endocarditis, one both upper and lower limb, two torso, three urine cultures, one peritoneal dialysis fluid, one ear swab, two eye swabs.

^d^ incomplete clinical information.

There were 22 episodes of invasive *Pasteurella* spp. infections, including pulmonary infection (11; 50%), septic arthritis (3; 13.6%), osteomyelitis (2; 9.1%), diabetic foot infection with osteomyelitis (2; 9.1%), prepatellar bursitis (1; 4.5%), head and neck infection with cervical lymphadenitis (1; 4.5%), peritoneal dialysis-related peritonitis (1; 4.5%) and blepharitis following penetrating eye trauma (1; 4.5%). Adults aged 16 to 65 years had the majority of invasive infections (16, 72.9%).

The majority of *Pasteurella* spp. infections were localised, with superficial local infections (98; 50.2%) outnumbering deep local infections (34; 17.4%). Unlike bloodstream and invasive infections, there was a female predominance in deep and superficial local infections, accounting for 58.8% and 57.1% respectively. Deep local infections, including abscesses (16; 47.1%), necrotic soft tissue infections (12; 35.2%), penetrating chest wounds with deep purulent material (2; 5.9%), pyomyositis (1; 2.9%) and tenosynovitis (1; 2.9%), were more common with lower limb wounds (22, 64.7%). Superficial infections were more common in upper limbs (48, 49.0%), and consisted of localised bite wound infection (59; 60.2%) and cellulitis (36; 36.7%); three had incomplete clinical information.

Infections in the ‘other’ category included otitis media (1; 20%), conjunctivitis (1; 20%), and three positive urine cultures with incomplete clinical information.

Underlying comorbidities were frequently reported (99 of 173; 57.1%) overall, but more so in those with bloodstream infections (20 of 21; 95.2%), invasive infections (14 of 19; 73.7%), and deep localised infections (21 of 33; 63.6%), than in those with superficial infections (56 of 96; 58.3%). Patients with bacteraemia had underlying malignancy (5; 22.7%), diabetes mellitus (4; 18.2%), renal disease (4; 18.2%) and cirrhosis (2; 9.1%). Patients with invasive disease had diabetes mellitus (4; 18.2%), renal disease (4; 18.2%), malignancy (2; 9.1%), chronic pulmonary obstructive disease (2; 9.1%), other respiratory disorders (2; 9.1%), cirrhosis (1; 4.5%) and autoimmune disease (1; 4.5%). There was a trend for male patients to be overrepresented in the bloodstream and invasive infection groups, but did not reach statistical significance (*p = 0*.*071)*.

Animal contact was documented in 145 (74.4%) episodes – dog bites were most common (82; 42.1%), followed by cat bites or scratches (62; 31.8%), and one child who was gored by a pig. Patients with bloodstream infection most frequently reported cat contact (8, 36.4%). In those with invasive infections, preceding dog bites were documented in a few (4; 18.2%), although the majority had no animal contact documented in clinical notes. This was the case for all sputum isolates, and the peritoneal dialysis fluid. Dog exposure was more commonly reported in localised infections, both deep (15; 44.1%) and superficial (53; 54.1%). When site of infection was compared, upper limb infections occurred more commonly after cat contact (33; 53.2%) than dog bites (26; 31.7%). Conversely, patients with lower limb infections reported more dog bites (46; 56.1%) than cat bites or scratches (26; 31.7%). Patients with head and neck infections reported more dog bites (6; 75%) than cat contact (1; 12.5%); four of these cases were facial dog bites in children.

Clinical management and outcomes are summarised in Table 3. Median length of stay was longer for bloodstream and invasive infections than for other infections (*p < 0*.*001*). Surgical debridement was required in 82 of 181 patients (45.3%) overall; this was more likely to happen in deep localised infections (29 of 33; 87.9%), twelve of which had necrotic tissue present at debridement. Fourteen episodes had incomplete clinical information regarding surgery. Paediatric patients were more likely to receive surgical debridement (13 of 21; 61.9%) than adults (69 of 160; 43.1%), *p = 0*.*004*.

**Table 3:** Management and clinical outcomes of patients with *Pasteurella* spp. infections.

	Bloodstream (n = 22)	Invasive (n = 22)	Deep local (n = 34)	Superficial local (n = 98)	Other ^a^ (n = 5)	Unknown (n = 14)	Total (n = 195)	P value
**Admission to hospital**	22 (100.0)	15/21 (71.4) ^b^	34 (100.0)	72 (73.5)	2/4 (50.0) ^b^	3/14 (2.1)	148/193 (76.7) ^b^	*P < 0*.001 *
**Length of stay of admitted patients, median (IQR)**	7.0 (5.0 – 12.0)	9.0 (3.5 – 29.5)	3.0 (2.0 – 5.8)	2.0 (2.0 -4.0)	0.5	1.0 (0.5 – 1.5)	3.0 (2.0 – 6.0)	*P < 0*.001 *
**Duration of intravenous antibiotic therapy, median (IQR)**	7.0 (4.5 – 11.0) ^c^	2.0 (0.0 – 6.0) ^c^	3.0 (2.0 – 5.0) ^c^	2.0 (0.5 – 3.3) ^c^	-	-	3.0 (1.0 – 5.0) ^c^	*P < 0*.001 *
**Duration of oral antibiotic therapy,** **median (IQR)**	10.0 (6.0 – 14.0) ^c^	7.0 (5.0 – 14.0) ^c^	7.0 (5.0 – 7.0) ^c^	5.0 (5.0 – 7.0) ^c^	-	-	7.0 (5.0 – 8.0) ^c^	*P = 0*.02 *
**Total duration of antibiotic therapy, median (IQR)**	17.0 (13.0 – 25.5) ^c^	9.0 (7.0 – 28.0) ^c^	10 (7.0 – 12.0) ^c^	8.0 (6.0 – 10.0) ^c^	-	-	9.5 (7.0 – 12.3) ^c^	*P < 0*.001 *
**Surgery**	5/20 (26.3) ^b^	7/18 (38.9) ^b^	29/33 (87.9) ^b^	41/95 (43.2) ^b^	0	0	82/181 (45.3) ^b^	*P < 0*.001 *
**ICU admission**	2 (9.1)	0	0	1 (1.0)	0	0	3 (1.5)	*P = 0*.*101*
**Mortality at 30 days**	1 (4.5)	0	0	1 (1.0)	0	0	2 (1.0)	*P = 0*.*650*

^a^ includes three urine cultures, one ear swab and one eye swab

^b^ number of complete clinical records as demoninator

^c^ based on 116 episodes where complete prescription data was available.

Complete antibiotic prescribing data was available for 116 episodes. Median duration of intravenous antibiotic therapy and total antibiotic therapy were longer in patients with bloodstream infections. Beta-lactam with beta-lactamase inhibitors (BLBLI) were the most frequently prescribed antibiotics. Amoxicillin-clavulanate was the most common oral prescription (82; 50.9%), as well as the most common intravenous prescription (24; 20.7%) from 2019 when it became available in the health service. Prior to this, the most frequent intravenous BLBLI presriptions were piperacillin-tazobactam (45; 38.8%) and ticarcillin-clavulanate (6; 5.2%) prior to 2013. Other intravenous antibiotics prescribed included benzylpenicillin, ampicillin, flucloxacillin, cephazolin, ceftriaxone, meropenem, metronidazole, gentamicin, doxycycline, vancomycin, lincomycin, clindamycin and ciprofloxacin. Other oral antibiotics prescribed included amoxicillin, phenoxymethylpenicillin, dicloxacillin, cefalexin, doxycycline, metronidazole, clindamycin, ciprofloxacin and trimethoprim-sulfamethoxazole. One patient with blepharitis was treated with topical chloramphenicol. One patient with a superficial wound infection did not receive any antibiotic therapy.

Overall, admission to the intensive care unit (3; 1.5%) and all-cause 30-day mortality were low (2; 1.0%). When analysing those with severe infections only (bacteraemia and invasive infections), mortality remained low (1 of 44; 2.3%).

### Microbiology

As summarised in Table 4, *Pasteurella multocida* was the most commonly isolated *Pasteurella* spp. (121; 61.1%), followed by *P*. *canis* (45; 23.1%) and *P*. *dagmatis* (2; 1.0%). 30 (15.4%) isolates were reported as *Pasteurella* species (not otherwise specified), as were not able to be further identified to the species level. Three patients had two *Pasteurella* spp. isolated – *P*. *multocida* and *Pasteurella* species (not otherwise specified)*; P*. *multocida* and *P*. *dagmatis*; and *P*. *canis* and *P*. *dagmatis*.

**Table 4:** Microbiology of *Pasteurella* spp. infections.

*Pasteurella* species	Number (% specimens)
*Pasteurella multocida*	121 (61.1)
*Pasteurella canis*	45 (23.1)
*Pasteurella dagmatis*	2 (1.0)
*Pasteurella* species, not otherwise specified	30 (15.4)
**Polymicrobial**	**132 (67.7)**
**Specimens with significant organisms isolated**	**45 (23.1)**
** Gram-positive**	
** ** *Staphylococcus aureus*	23 (11.8)
** ** *Streptococcus pyogenes*	7 (3.6)
** ** *Streptococcus C*	2 (1.0)
** ** *Streptococcus G*	7 (3.6)
** ** *Streptococcus suis*	1 (0.5)
** ** *Streptococcus pneumoniae*	1 (0.5)
** ** *Arcanobacterium haemolyticum*	3 (1.5)
** ** *Clostridium septicum*	1 (0.5)
** Gram-negative**	
** ***Aeromonas sp*.	1 (0.5)
** ***Alcaligenes sp*.	1 (0.5)
** ** *Citrobacter koseri*	1 (0.5)
** ** *Enterobacter cloacae*	2 (1.0)
** ** *Haemophilus influenzae*	3 (1.5)
** ** *Moraxella catarrhalis*	1 (0.5)
** ** *Morganella morganii*	1 (0.5)
** ** *Neisseria animaloris/zoodegmatis*	2 (1.0)
** ** *Pseudomonas aeruginosa*	6 (3.1)
** ***Pseudomonas sp*.	1 (0.5)
** ** *Vibrio alginolyticus*	1 (0.5)
** Yeast**	
** ** *Candida tropicalis*	1 (0.5)

Polymicrobial defined as multiple organisms seen on Gram stain or culture; where not specified further, included mixed cutaneous flora, mixed oral flora and mixed anaerobes. Three specimens had more than one *Pasteurella* species isolated (*P*. *multocida* and *P*. *species*, *P*. *dagmatis* and *P*. *canis*, *P*. *dagmatis* and *P*. *multocida*).

The majority of bloodstream isolates were *P*. *multocida (20; 90*.*9%)*, with one each of *P*. *canis* and *Pasteurella* species (not otherwise specified). In the invasive infections, *P*. *multocida* also predominated (15; 68.2%), followed by *P*. *canis* (4; 18.2%) and *Pasteurella* species (not otherwise specified) (4, 18.2%); one head and neck infection isolated both *P*. *multocida* and *Pasteurella* species (not otherwise specified).

Patients with cat contact isolated *P*. *multocida* (57; 90.4%), *P*. *dagmatis* (1; 1.6%) and *Pasteurella* species (not otherwise specified) (5; 7.9%). Patients with dog bites isolated *P*. *canis* (35; 42.2%), *P*. *multocida* (30; 36.1%); *P*. *dagmatis* (1; 1.2%) and *Pasteurella* species (not otherwise specified) (17; 20.5%). The single isolate from pig exposure was *Pasteurella* species (not otherwise specified). Of the specimens in which animal contact was not reported, identified isolates were *P*. *multocida* (34; 66.7%), *P*. *canis* (10; 19.6%) and *Pasteurella* species (not otherwise specified) (7; 13.7%).

The majority of specimens were polymicrobial (132; 67.7%). *Staphylococcus aureus* was the most frequent co-infection (23; 11.8%), followed by *Streptococcus pyogenes* (7; 3.6%), Group G Streptococcus (7; 3.6%), *Pseudomonas aeruginosa* (6; 3.1%), *Haemophilus influenzae* (3; 1.5%) and *Arcanobacterium haemolyticum* (3; 1.5%). Three of the *S*. *aureus* isolates were non-multiresistant *methicillin-resistant S*. *aureus* (nmMRSA); the rest were methicillin susceptible (MSSA). The majority of polymicrobial infections had mixed anaerobes, cutaneous or enteric flora.

## Discussion

Though *Pasteurella* spp. were described by Pasteur over 140 years ago [18], this current study is one of the few to highlight the significant associated morbidity in a single location, and wide spectrum of *Pasteurella* spp. infections presenting to a tertiary hospital. This study’s findings of note were: (i) the low mortality in bacteraemic and invasive infection patients with laboratory diagnosed infections compared to other studies; (ii) the increasing number and varied presentations to the hospital system in recent years; and (iii) the polymicrobial nature of bite infections which has implications for prophylactic antibiotic therapy.

Forty-four patient infections (22.6%) in this study were identified as having bloodstream or other invasive infections. A recent French study of *Pasteurella* spp. infections over 14 years found similar numbers, with 45 of 215 (20.9%) infections to be bloodstream or invasive infections, including 14 (6.5%) bloodstream, 17 (7.9%) pulmonary and 14 other invasive, including osteoarticular, intra-abdominal, ocular and central nervous system infections [11]. A retrospective study in Israel found 28.6% (25 of 77) of cases had bacteraemia, although relied on a questionnaire completed by only 57.1% of contacted hospitals, so may not reflect the true incidence of bacteraemia in Israel.

Patients with bacteraemia were likely to be older, consistent with another Australian study, which found a marked increase in incidence with advancing age [2]. Invasive, deep and superficial infections were more common in the 16 to 65 year age group, consistent with population.

Co-morbidities were present in 57.2% of patients overall, and there was a particular association between underlying comorbidities and bloodstream infections (95.2%). Comorbidities were least common in the superficial infection group, suggesting that underlying health conditions predispose to more severe disease. Previous papers have reported commonly identified comorbidities of diabetes mellitus (reported rates of 14% to 34%) [2,10–12], cirrhosis (24%) [10], congestive heart failure (9% to 21%) [2,10], renal disease (6% to 19%) [2,10,11], chronic obstructive pulmonary disorder (9%) [11] and autoimmune conditions (3% to 7%) [10,11], highlighting a broad spectrum of clinical conditions that alter the host response to infection, and therefore risk for bacteraemia and invasive disease [2,10,11].

There was a low overall mortality rate at 1% in our study. When comparing mortality from bacteraemia alone (4.5%) or combined bacteraemia and invasive infection (2.3%), this study still had much lower rates than previous literature (8% to 31%) [2,10,12]. This is despite high rates of comorbidities, and a comparable median age to the only other Australian study – Laupland *et al*. found a median age of 75.0 years (IQR 64.7 – 83.4 years), with a mortality rate of 8% [2]. Lower mortality rates may be reflective of the smaller number of bloodstream infections seen, or the longitudinal study design with less risk of selective bias.

We observed a wide spectrum of disease. Osteomyelitis, septic arthritis and pneumonia have been previously reported [19,20] as well as peritoneal dialysis-related peritonitis [21] in case reports only. Local infections such as cellulitis and localised wound infections still account for the majority of *Pasteurella* spp. infections, however the preponderance for necrotising soft tissue disease and abscess formation cannot be discounted. Two cases of conjunctivitis were noted in this study – this has previously been described in adult patients with known animal contact [22]. There was also one case of otitis media, which is not commonly reported, although a review of *Pasteurella* spp. meningitis found 25% of published cases had associated otitis media with suspected contiguous spread [23]. There were no cases of meningitis in the current study.

In our study, lower limb infections were more common in the bloodstream, invasive and deep infection groups, with upper limb infections only being more common in the superficial groups. A possible explanation may be earlier healthcare seeking behaviour following an animal bite involving the hands.

Paediatric animal bite wounds are more likely to require surgical intervention, including general anaesthesia for adequate debridement and primary closure [8]. This was evident in the current study, with 61.9% receiving surgical debridement. Children are more likely to receive animal bites to the head and neck [8,9], due to patient size and behaviours of kissing and hugging dogs [9]. There were four dog bites to the face in this cohort, all of which required admission and surgical debridement. A larger study on dog bites in children found 320 (71%) bites to the face, head and neck required admission, compared to 128 (29%) that were managed in the emergency department alone [9].

This study has highlighted the polymicrobial nature of animal bites with 23.8% episodes also isolating another significant pathogen, half of which were *Staphylococcus aureus*. Interestingly, only three (13%) isolates in the cohort had nmMRSA, lower than national (17.6%, AGAR), and regional (36%, antibiogram) rates of nmMRSA. Therapeutic guidelines currently recommend empiric amoxicillin-clavulanate for bite-associated wounds, or a combination of metronidazole and doxycycline in areas of high nmMRSA prevalence [24]; while appropriate for the majority of isolates found in this study, 3.1% of infections also included *P*. *aeruginosa*, revealing the benefit of specimen culture. The widespread use of empiric antibiotics does not seem to have decreased hospital presentations or actual number of infections, as our study has demonstrated increased laboratory confirmation over a 20 year period.

Similar to the recent publication on *Pasteurella* spp. bloodstream infections in Australia [2], the incidence of laboratory-confirmed *Pasteurella* spp. infections was found to increase over the course of this study. It is unclear whether there is a true increase in incidence, or increasing presentations. Possible reasons include increased general-practitioner type presentations to emergency departments [25,26], increased clinical awareness in collecting specimens from animal bite injuries, and improved laboratory diagnostic methods.

The geographic distribution of *Pasteurella* spp. infections was largely around urban areas, reflecting population density, and therefore pet ownership. Dog and cat ownership is common in Australia, and in urban centres are often registered under municipality jurisdiction. However, animals in remote communities in Australia are numerous and much less likely to be registered. A study of 20 remote communities in the Northern Territory found a median of 76.5 dogs and 3.0 cats per community, equivalent to approximately 6.3-fold higher dog ownership and similar cat ownership when compared to the average of Australian households [27]. Despite this, Aboriginal people did not have higher than expected rates of hospital presentations due to *Pasteurella* spp. infections. Some remote communities in the Top End utilise private interstate laboratory services, which may explain this. Another possible explanation is early prophylactic antibiotic prescription by community health centres, which is postulated to reduce those requiring transfer to the public health system in the regional centres.

Only one previous study found a predominance of infections in the spring and summer months [12]. There were no seasonal differences detected in this study, although is in a tropical climate.

The limitations of the study are the retrospective nature resulting in incomplete or no clinical information documented in the electronic medical record, and therefore unknown animal contact for a quarter of cases. Secondly, the relatively small size of our cohort of invasive and bacteraemic infections, which may mean our conclusions have wide confidence intervals and findings may not be generalised.

*Pasteurella* spp. remain clinically important pathogens in clinical practice, with the ability to cause severe and invasive infections. Presentations of bite wounds to hospital settings are becoming more common, and associated morbidity and mortality remains significant. The polymicrobial nature of bite wounds has been demonstrated and this has implications for prophylactic post exposure antimicrobial prescription.
